# Blood pressure and heart rate changes during shifts in ICU nurses in relation to their work experience

**DOI:** 10.1186/cc14586

**Published:** 2015-03-16

**Authors:** A Ioannidis, E Terzenidou, D Gklava, I Politi, E Georgiadou, A Georgousi, V Aidonoudis, A Kalea, P Melitzana, N Gritsi-Gerogianni

**Affiliations:** 1 Thessaloniki General Hospital 'Ippokratio', Thessaloniki, Greece

## Introduction

The aim of the study was to assess the blood pressure (BP) and heart rate (HR) changes during shifts in ICU nurses in relation to their work experience. Our hypothesis was that less experienced nurses, in comparison with more experienced ones, would be subjected to more work stress and this could be demonstrated by higher changes in BP and HR during shifts.

## Methods

We enrolled 23 nurses working in an 8-hour shift schedule at a general adult ICU. Demographic and clinical data were obtained by completing a short questionnaire. The nurses were invited to measure their BP and HR at the beginning, in the middle and at the end of their shift. An ESH/BSH-certified automatic device was used for the BP and HR measurements.

## Results

The mean duration of working in an ICU was 7.3 ± 5.32 years (from 2 to 18 years). The nurses were grouped according to experience - Group A: 17 nurses with <10 years of experience (mean: 4.4 years), Group B: six nurses with >10 years (mean: 15.6 years). There were 640 BP-HR measurements. The mean systolic BP, diastolic BP and HR did not differ between the two groups (systolic BP: 111.2 ± 10.9 vs. 113.7 ± 14.1 mmHg, *P *= 0.654; diastolic BP: 72.8 ± 8.2 vs. 71.9 ± 8.1 mmHg, *P *= 0.835; HR: 81.4 ± 7.2 vs. 78.1 ± 8.4 bpm, *P = *0.365). Nevertheless, the mean change in BP and HR during the shift did differ between the two groups, with the more experienced nurses showing a trivial reduction in systolic and diastolic BP and minor increase in HR whereas the less experienced ones showed slight increase in both BP and HR measurements (Table 1), reaching almost statistical significance. For the less experienced nurses in Group 1, it was noted that the mean changes were bigger in night shifts although the limited number of measurements did not allow robust statistical analysis.

**Table 1:** Mean change in systolic and diastolic BP and HR in relation to ICU work experience.

	Group 1	Group 2	*P *value
SBP (mmHg)	3.22	-0.40	0.053
DBP (mmHg)	2.08	-0.27	0.061
HR (bpm)	4.46	1.16	0.48

## Conclusion

The less experienced ICU nurses, with <10 years of ICU work experience, tended to increase their BP and HR levels during the shift, a finding probably heightened during night shifts. Further research, including not only cardiovascular parameters, is warranted to uncover the effects of shift-work pattern in ICU nurses, taking into account this specifically stressful work environment.

